# Critical causes in severe bleeding requiring angioembolization after percutaneous nephrolithotomy

**DOI:** 10.1186/s12894-020-00594-6

**Published:** 2020-03-11

**Authors:** Hee Youn Kim, Kyu Won Lee, Dong Sup Lee

**Affiliations:** 1 Department of Urology, St. Vincent’s Hospital, College of Medicine, The Catholic University of Korea, 93, Jungbu-daero, Paldal-gu, Suwon-si, Gyeonggi-do 16247 Republic of Korea; 2 Department of Urology, Seoul St. Mary’s Hospital, College of Medicine, The Catholic University of Korea, 222, Banpo-daero, Seocho-gu, Seoul, 06591 Republic of Korea

**Keywords:** Kidney calculi, Percutaneous nephrolithotomy, Hemorrhage, Therapeutic embolization

## Abstract

**Background:**

To identify the risk factors for severe bleeding requiring angioembolization among patients who received transfusions after PCNL, particularly those who underwent anatomically incorrect renal puncture.

**Methods:**

A total of 53 patients, who received transfusions after PCNL and simultaneously had a postoperative CT scan performed between November 2009 and May 2019 at two teaching hospitals, were retrospectively reviewed. The patients were divided into two groups: those who underwent angioembolization and those who did not. Patient, stone and procedural factors were compared between the two groups. Puncture correctness was evaluated using postoperative CT scans. Puncture was defined as being a correct puncture if the fornix or papilla of the posterior calyx was punctured and the trajectory of the tract was within 20 degrees posterior to the frontal plane of the kidney (i.e., within Brödel’s line).

**Results:**

21 patients underwent angioembolization after PCNL. Incorrect puncture was seen in 14/21 (66.7%) patients who underwent angioembolization after PCNL, whereas it was seen in 11/32 (34.4%) patients who did not undergo angioembolization (*p* = 0.021). On multivariable regression analysis, puncture correctness was found to be the only significant factor, with an OR of 3.818, 95% CI of 1.192–12.231 and *p* value of 0.024.

**Conclusions:**

Incorrect renal puncture was related to severe bleeding requiring angioembolization after PCNL. Our results emphasize the importance of the basic principle of renal puncture for PCNL.

## Background

Anatomical understanding of the pelvocalyceal system and the related renal vasculature is essential to prevent bleeding complications during percutaneous nephrolithotomy (PCNL). Brödel’s line of bloodless incision, the relatively avascular plane where the anterior and posterior segmental renal artery branches meet, is the key anatomical area [1]. Historically, before the era of PCNL, when anatrophic nephrolithotomy was performed, incision of the kidney was performed through this Brödel’s line to minimize bleeding. The same principle applies to the PCNL procedure. The puncture should ideally traverse the relatively avascular Brödel’s line, thereby decreasing the risk of bleeding. This is why the posterior calyx is considered the optimal calyx to puncture because posterior calices are usually oriented towards Brödel’s line [2–4].

When the puncture is made outside of Brödel’s line, the risk of arterial injuries increases, potentially leading to persistent and severe bleeding. Although rare, injury to these arteries can cause hemodynamic instability and can be potentially life-threatening. A high degree of suspicion, prompt angiography and subsequent angioembolization are required in these instances.

Past reports that studied risk factors for angioembolization after PCNL did not consider whether anatomically correct puncture was performed [5, 6]. In the current study, we aimed to identify risk factors of severe bleeding requiring angioembolization among patients who received transfusions after PCNL, particularly those involved in anatomically correct renal puncture (i.e., through Brödel’s line).

## Methods

### Study population and design

**Fig. 1:**
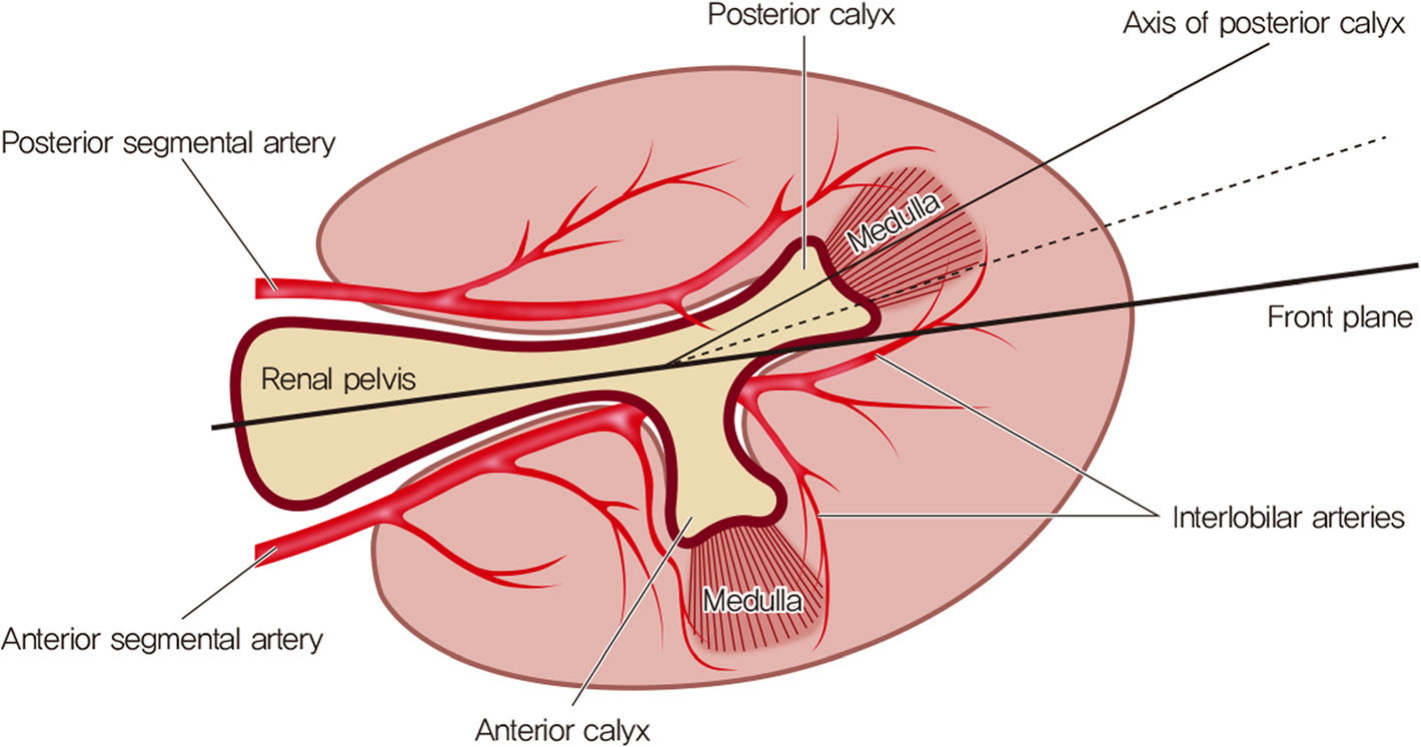
Anatomy of axial view of kidney. Puncture was defined as being a correct puncture if the fornix or papilla of the posterior calyx was punctured and the trajectory of the tract was within 20 degrees posterior to the frontal plane of the kidney (i.e., within Brödel’s line)

**Fig. 2:**
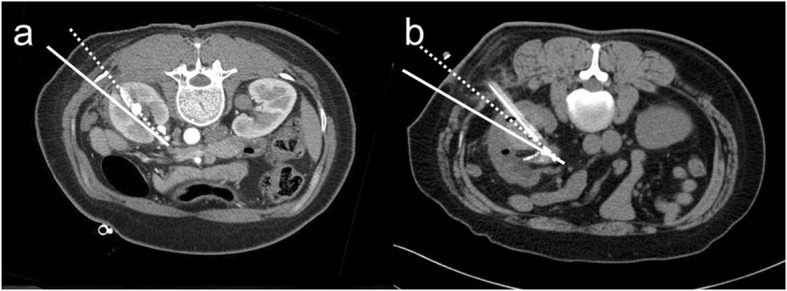
Example of a CT scan of a correct (**a**) and incorrect (**b**) puncture. Solid line represents the frontal plane, and dotted line represents the line 20 degrees posterior to the frontal plane. In the CT scan of correct puncture (**a**), the tract is punctured within Brödel’s line. In the CT scan of incorrect puncture (**b**), the tract is punctured outside of Brödel’s line

The current study used a retrospective design. A chart review of all patients who received transfusions after PCNL between November 2009 and May 2019 at two teaching hospitals was performed. Among these patients, only those who had undergone a postoperative computer tomography (CT) scan to evaluate the cause of bleeding were included. Patients with kidney anomalies (e.g., horseshoe kidney, calyceal diverticulum, etc.), and patients who underwent simultaneous bilateral procedures were excluded. The included patients were divided into two groups: patients who did not undergo angioembolization (No AE) and patients who underwent angioembolization (AE). Patient, stone and procedure-related characteristics were recorded and compared between the two groups. Patient characteristics included age, sex, body mass index (BMI) and American Society of Anesthesiologists (ASA) classification. Stone characteristics included laterality, stone location, stone size, staghorn stone, Guy’s stone score [7], Hounsfield unit, degree of hydronephrosis, and preoperative percutaneous nephrostomy. The degree of hydronephrosis was evaluated using the criteria of the Society for Fetal Urology (SFU) [8]. SFU grade 0 was defined as ‘no hydronephrosis’, SFU grades I-III was defined as ‘mild hydronephrosis’, and SFU grade IV was defined as ‘severe hydronephrosis’. Procedure-related characteristics included operation time, location of access (lower pole, other (middle or upper), and lower pole plus other), number of tracts, puncture correctness and stone free rate. Puncture correctness was evaluated using postoperative CT scans. Puncture was deemed a correct puncture if the fornix or papilla of the posterior calyx was punctured and the trajectory of the tract was within 20 degrees posterior to the frontal plane of the kidney (i.e., within Brödel’s line) (Figs. 1 and 2) [2–4]. Stone free rate was evaluated with postoperative CT scans within months. Residual fragments under 2 mm was considered insignificant. Details of the angioembolization were also reviewed. The interval between the surgery and angioembolization, and the angiographic findings were recorded.

### Surgical technique

Here, the PCNL procedure is briefly described. A ureteral occlusion balloon catheter was inserted via a cystoscope. All PCNL procedures were performed in the prone position with fluoroscopic assistance. A balloon dilatator (X-FORCE^Ⓡ^ N30 Nephrostomy Balloon Dilation Catheter, Bard Medical, GA, USA) was used for tract dilatation, and a 30F Amplatz sheath was used. A rigid 24F nephroscope (Richard Wolf, Knittlingen, Germany) was used for stone fragmentation and extraction. An antegrade ureteral catheter and a 20F nephrostomy tube were placed at the end of the operation.

### Data analysis

SPSS (IBM Corp. Released 2012. IBM SPSS Statistics for Windows, Version 21.0. Armonk, NY: IBM Corp.) was used for the statistical analysis. The comparison of continuous variables was performed using the unpaired t-test or the Mann-Whitney test based on the result of the Shapiro-Wilk test for normality. The comparison of categorical variables was performed using the chi-square test or Fisher’s exact test. A multivariate logistic regression analysis was conducted to investigate the risk factors of angioembolization. A *p* value < 0.05 was considered significant.

## Results

The baseline patient and stone characteristics of the two groups are described in Table 1. Of the 1554 patients who underwent PCNL during the study period, 53 patients received blood transfusion after the surgery and simultaneously underwent a postoperative CT scan. 32 patients did not undergo angioembolization (No AE group) and 21 underwent angioembolization (AE group). The rate of angioembolization after PCNL was 1.4% (21/1554). There was no difference in age, BMI or ASA classification between the two groups. A statistically significant male predominance was noted in the AE group (43.8% versus 71.4%, *p* = 0.048). There was no difference in stone laterality, stone location, stone size, presence of staghorn stone, Guy’s stone score, Hounsfield unit, degree of hydronephrosis, or preoperative PCN.

**Table 1:** Comparison of baseline information between patients who did not undergo angioembolization and patients who underwent angioembolization after percutaneous nephrolithotomy

	No AE	AE	*p* value
Number of cases	32	21	
Sex			0.048^a^
Male	14/32 (43.8%)	15/21 (71.4%)	
Female	18/32 (56.3%)	6/21 (28.6%)	
Age (years)	58.9 ± 12.2	61.3 ± 13.9	0.511^b^
BMI (kg/m^2^)	23.6 ± 3.3	23.6 ± 3.8	0.977^b^
ASA classification			0.371^a^
Class I	7/32 (21.9%)	6/21 (28.6%)	
Class II	25/32 (78.1%)	14/21 (66.7%)	
Class III	0/32 (0.0%)	1/21 (4.8%)	
Class IV	0/32 (0.0%)	0/21 (0.0%)	
Stone laterality			0.538^a^
Right	14/32 (43.8%)	11/21 (52.4%)	
Left	18/32 (56.3%)	10/21 (47.6%)	
Stone location			0.124^c^
Upper	0/32 (0.0%)	0/21 (0.0%)	
Middle	1/32 (3.1%)	0/21 (0.0%)	
Lower	2/32 (6.3%)	3/21 (14.3%)	
Pelvis	3/32 (9.4%)	6/21 (28.6%)	
Multiple	26/32 (81.3%)	12/21 (57.1%)	
Stone size (cm)	3.2 ± 1.5	3.3 ± 1.6	0.927^d^
Staghorn stone	19/32 (59.4%)	9/21 (42.9%)	0.272^a^
Guy’s stone score			0.423^a^
Grade I	3/32 (9.4%)	5/21 (23.8%)	
Grade II	10/32 (31.3%)	5/21 (23.8%)	
Grade III	15/32 (46.9%)	10/21 (47.6%)	
Grade IV	4/32 (12.5%)	1/21 (4.8%)	
Hounsfield unit	976 ± 265	1103 ± 369	0.150^b^
Hydronephrosis			0.615^a^
None	8/32 (25.0%)	5/21 (23.8%)	
Mild	23/32 (71.9%)	14/21 (66.7%)	
Severe	1/32 (3.1%)	2 /21 (9.5%)	
Pre-operative PCN			0.374^c^
Not done	30/32 (93.8%)	18/21 (85.7%)	
Done	2/32 (6.3%)	3/21 (14.3%)	

*AE* angioembolization, *ASA* American Society of Anesthesiologists, *UPJ* ureteropelvic junction, *IPA* infundibulopelvic angle, *PCN* percutaneous nephrostomy

Results of continuous variables expressed as mean ± standard deviation

^a^Chi-square

^b^Unpaired t-test

^c^Fisher’s exact test

^d^Mann Whitney test

Procedure-related characteristics are described in Table 2. There was no difference in operation time, location of access, number of tracts and stone free rate between the two groups. Puncture correctness showed a statistically significant difference, where correct puncture was noted in 65.6% of the No AE group and 33.3% of the AE group (*p* = 0.021). Incorrect puncture in patients who underwent angioembolization occurred approximately twice as often as in patients who did not undergo angioembolization.

**Table 2:** Comparison of procedural details between patients who did not undergo angioembolization and patients who underwent angioembolization after percutaneous nephrolithotomy

	No AE	AE	*p* value
Operation time (minutes)	114.0 ± 55.8	90.4 ± 34.5	0.187^a^
Location of access			0.266^b^
Lower pole	20/32 (62.5%)	17/21 (81.0%)	
Other (middle or upper)	11/32 (34.4%)	3/21 (14.3%)	
Lower pole & other	1/32 (3.1%)	1/21 (4.8%)	
Number of tracts			1.000^c^
1	31/32 (96.9%)	20/21 (95.2%)	
2	1/32 (3.1%)	1/21 (4.8%)	
Puncture correctness			0.021^b^
Correct	21/32 (65.6%)	7/21 (33.3%)	
Incorrect	11/32 (34.4%)	14/21 (66.7%)	
Stone free rate	24/32 (75.0%)	15/21 (71.4%)	1.000^b^
Interval between surgery and angioembolization (days)	NR	7.4 ± 6.4	
Angiographic findings	NR		
Pseudoaneurysm		15/21 (71.4%)	
AVF		5/21 (23.8%)	
Both		1/21 (4.8%)	

*AE* angioembolization, *UPJ* ureteropelvic junction, *AVF* arteriovenous fistula, *NR* not relevant

Results of continuous variables expressed as mean ± standard deviation

^a^Mann-Whitney U test

^b^Chi-square test

^c^Fisher’s exact test

Multivariable logistic regression analysis was performed to identify predictive factors of angioembolization after percutaneous nephrolithotomy and the results are shown in Table 3. Puncture correctness was the only factor that showed statistical significance, with an odds ratio (OR) of 3.818, 95% confidence interval (CI) of 1.192–12.231 and *p* value of 0.024.

**Table 3:** Multivariable logistic regression analysis to find out predictive factors of angioembolization after percutaneous nephrolithotomy in patients who received transfusion

	OR	95% CI	*p* value
Puncture correctness	3.818	1.192–12.231	0.024

*OR* odds ratio, *CI* confidence interval, *UPJ* ureteropelvic junction

## Discussion

Despite the increasing transition to a less invasive surgical method for removal of kidney stones such as retrograde intrarenal surgery in recent years, percutaneous nephrolithotomy (PCNL) is still an integral part of treatment for large kidney stones [9]. Through decades of surgical experience with PCNL since it gained popularity after Fernström and Johannson’s report in 1976 [10], PCNL has proven to be an effective surgery for kidney stone removal.

Nevertheless, the reported complication rate of PCNL, although lower than the open approach, is relatively high. Bleeding is the most common and significant complication of PCNL, with the reported incidence of bleeding requiring transfusion as high as 20%, with 7% as the average incidence [11]. Most bleeding complications result from venous injuries, which improve with conservative care such as transfusion and usually do not cause hemodynamic instability. However, when the cause of bleeding is arterial injuries, persistent and severe bleeding may occur that does not improve with transfusion alone, leading to hemodynamic instability. Prompt angiography and angioembolization are needed in these cases. Although the reported incidence of angioembolization after PCNL is not high, ranging between 0 and 1.5% [11], it can potentially be life-threatening if prompt measures are not undertaken. This retrospective study was conducted to determine what separates patients who suffer severe bleeding requiring angioembolization after PCNL from patients who bleed enough to receive transfusion but recover without further measures.

Several past studies have attempted to identify factors involved in angioembolization after PCNL. Srivastava et al. retrospectively analyzed 1854 patients and identified 27 (1.4%) patients who required angiography [6]. In the multivariate analysis, stone size was the only significant factor predicting the occurrence of bleeding complications requiring angioembolization. El-Nahas et al. retrospectively analyzed 39 of 3878 PCNL procedures that required angioembolization [5]. Their multivariate analysis identified upper calyceal puncture, solitary kidney, staghorn stone, multiple punctures and inexperienced surgeon as significant risk factors. The identified factors from these studies however, did not differ from known factors for bleeding in general after PCNL from many other studies [12–15]. This shows that studies fail to identify factors that specifically cause severe bleeding requiring angioembolization after PCNL, partly because they compare the results between patients who underwent angioembolization and patients who did not have any bleeding complications.

On this basis, the current study was designed to analyze risk factors of angioembolization after PCNL by dividing the patients who received transfusion after PCNL into two groups: those who underwent angioembolization and those who did not. In our study, the usual factors known for bleeding after PCNL from many other studies such as stone complexity, degree of hydronephrosis, operation time, etc., did not show significant difference between the two groups, implying that these factors may be risk factors for bleeding in general after PCNL, but they are not the decisive factors causing severe bleeding requiring angioembolization. Therefore, to identify the critical factor, we attempted to determine whether the puncture itself was performed correctly by examining postoperative CT scans of patients who received transfusions after PCNL. This, to the best of our knowledge, was not done in previous studies.

Establishing a proper renal access is the most crucial step in the PCNL procedure, but it is a process that is difficult to master. A surgeon has to mentally visualize the three dimensional anatomy of the pelvocalyceal system from two dimensional images obtained with CT scans or fluoroscope. Ideally, the fornix or papilla of the posterior calyx should be punctured and the trajectory of the tract should be within 20 degrees posterior to the frontal plane of the kidney. In the current study, incorrect puncture was seen in 14/21 (66.7%) patients who underwent angioembolization after PCNL, whereas it was seen in 11/21 (34.4%) patients who did not undergo angioembolization, which showed statistical significance (*p* = 0.021). On multivariable regression analysis, puncture correctness was found to be the only significant factor with an OR of 3.818, 95% CI of 1.192–12.231 and *p* value of 0.024. This result suggests that adhering to the basics of renal puncture is paramount in preventing severe bleeding after PCNL. Not only will this decrease the risk of severe bleeding by traversing the avascular plane of Brödel’s line, but it provides a relatively straight entry into the pelvis in the prone position [4]. If the fornix or papilla is missed and the infundibulum of the posterior calyx is punctured, injury to the interlobar arteries may occur [16]. Additionally, if the anterior calyx is punctured, the tract does not traverse Brödel’s line, leading to an increased risk of severe bleeding [16]. There will also be an acute angle between the tract and renal pelvis, leading to more torque and an increased chance of bleeding.

Recently, there were reports that suggested non-papillary or infundibular renal puncture was not associated with higher bleeding complications compared to papillary puncture [17]. However, these reports are from studies from single center and we feel that the evidence is not enough to suggest that non-calyceal or non-papillary puncture is as safe as its counterpart. There is also the issue of infundibular stricture. If the puncture is not done through the calyx or papilla and done through the infundibulum, deep injury to the surrounding tissue may lead to infundibular stricture [18, 19].

**Fig. 3:**
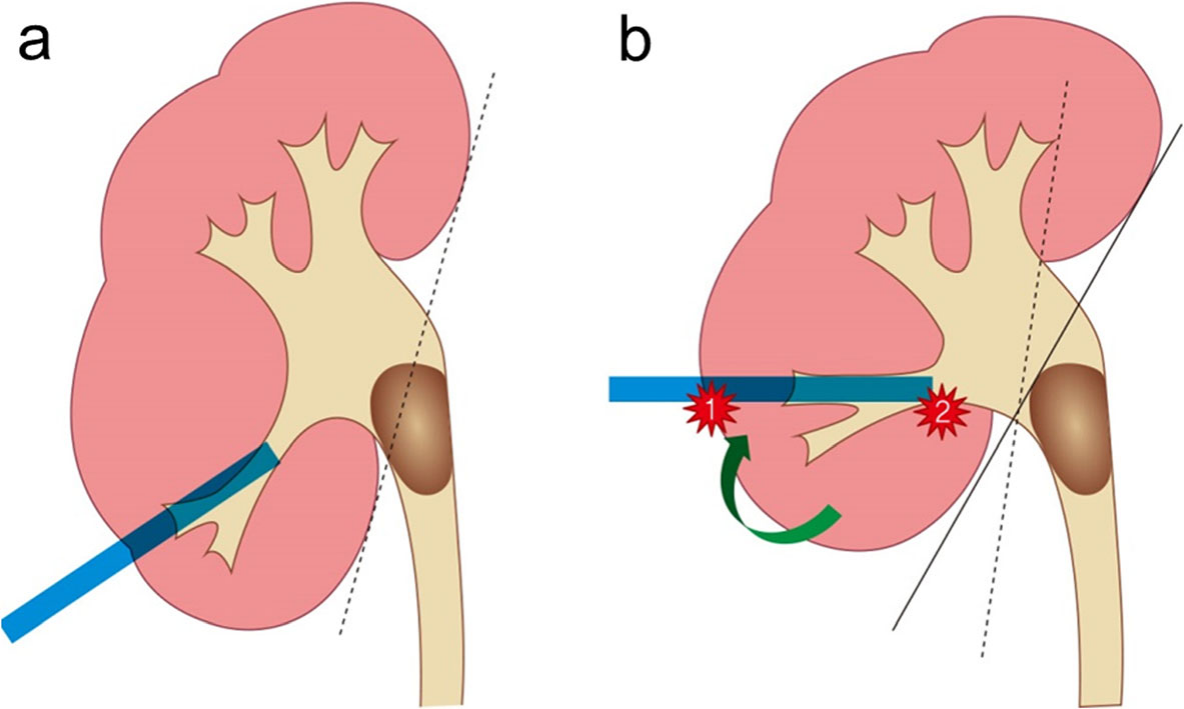
Renal pelvis stone with upper ureter extension approached by a lower pole calyceal approach (**a**). To approach the stone, excessive torque may be necessary which may cause bleeding (**b**)

There were 7 patients who, despite their puncture being correct on postoperative CT scans, underwent angioembolization. It was noted that a high proportion of these patients, 5 of 7 (71.4%), had a renal pelvis stone with extension into the upper ureter that was approached by the lower pole calyx. When selecting a pole for puncture, it is recommended that the pole that provides the most straight line along the stone axis be selected [20]. If this principle is not kept, the angle between the tract and the stone axis may become too acute, leading to excessive torque or a change in the direction of the tract, which can cause injury to the adjacent parenchyma with its vascular supply (Fig. 3) [20]. Some suggest that for best access to the ureteropelvic junction (UPJ), a pole whose calyx forms an angle of 90 degrees or more with the UPJ should be chosen [16]. When the angle was calculated for these 5 patients, it was 64.4 degrees, suggesting the possibility of excessive torque. If the middle or upper pole calyx was selected for renal puncture for these patients, bleeding could have been avoided. These results suggest that excessive torque may be one of the critical causes of severe bleeding leading to angioembolization after PCNL.

There were several limitations in the current study. First, the case number was relatively small, which was inevitable considering the very low incidence of severe bleeding requiring angioembolization after PCNL. Second, there was the possibility of selection bias because the study was retrospective. CT scans were not routinely performed for patients who received blood transfusion after PCNL. Patients who did not undergo a postoperative CT scan were excluded because of the study purpose. Third, we performed all of our PCNLs with patients in the prone position with fluoroscopic assistance using a 30F Amplatz sheath. Therefore, the results would not be applicable to PCNLs performed in the supine position, or PCNLs using smaller caliber sheaths, such as mini-PCNLs or ultramini-PCNLs.

## Conclusions

In the current study, we were able to identify whether the fornix or papilla of the posterior calyx was punctured and the trajectory of the tract was within Brödel’s line by examining postoperative CT scans of patients who received transfusions after PCNL. When this principle of anatomically correct renal puncture was not followed, the risk of severe bleeding requiring angioembolization after PCNL significantly increased. Our results emphasize the importance of the basic principle of renal puncture for PCNL.

## Data Availability

The datasets used and/or analyzed during the current study are available from the corresponding author on reasonable request.

## Abbreviations

AE: Angioembolization
ASA: American Society of Anesthesiologists
BMI: Body mass index
CI: Confidence interval
CT: Computed tomography
OR: Odds ratio
PCN: Percutaneous nephrostomy
PCNL: Percutaneous nephrolithotomy
SFU: Society for Fetal Urology
UPJ: Ureteropelvic junction
